# Risk prediction model establishment with tri-phasic CT image features for differential diagnosis of adrenal pheochromocytomas and lipid-poor adenomas: Grouping method

**DOI:** 10.3389/fendo.2022.925577

**Published:** 2022-12-08

**Authors:** Zhongfeng Niu, Jian Wang, Yang Yang, Jie He, Subo Wang, Zongyu Xie, Meihua Shao, Fangmei Zhu

**Affiliations:** ^1^ Department of Radiology, Sir Run Run Shaw Hospital, Zhejiang University School of Medicine, Hangzhou, Zhejiang, China; ^2^ Department of Radiology, Tongde Hospital of Zhejiang Province, Hangzhou, Zhejiang, China; ^3^ Department of Radiology, The First Affiliated Hospital of Bengbu Medical College, Bengbu, Anhui, China; ^4^ Department of Radiology, Shaoxing Hospital of Traditional Chinese Medicine, Shaoxing, Zhejiang, China

**Keywords:** computed tomography, adrenal, pheochromocytomas, adenomas, risk prediction model

## Abstract

**Objectives:**

The purpose of this study was to establish a risk prediction model for differential diagnosis of pheochromocytomas (PCCs) from lipid-poor adenomas (LPAs) using a grouping method based on tri-phasic CT image features.

**Methods:**

In this retrospective study, we enrolled patients that were assigned to a training set (136 PCCs and 183 LPAs) from two medical centers, along with an external independent validation set (30 PCCs and 54 LPAs) from another center. According to the attenuation values in unenhanced CT (CTu), the lesions were divided into three groups: group 1, 10 HU < CTu ≤ 25 HU; group 2, 25 HU < CTu ≤ 40 HU; and group 3, CTu > 40 HU. Quantitative and qualitative CT imaging features were calculated and evaluated. Univariate, ROC, and binary logistic regression analyses were applied to compare these features.

**Results:**

Cystic degeneration, CTu, and the peak value of enhancement in the arterial and venous phase (DEpeak) were independent risk factors for differential diagnosis of adrenal PCCs from LPAs. In all subjects (groups 1, 2, and 3), the model formula for the differentiation of PCCs was as follows: Y = -7.709 + 3.617*(cystic degeneration) + 0.175*(CTu ≥ 35.55 HU) + 0.068*(DEpeak ≥ 51.35 HU). ROC curves were drawn with an AUC of 0.95 (95% CI: 0.927–0.973) in the training set and 0.91 (95% CI: 0.860–0.929) in the external validation set.

**Conclusion:**

A reliable and practical prediction model for differential diagnosis of adrenal PCCs and LPAs was established using a grouping method.

## Introduction

Adrenal incidentalomas (AIs) were found to represent an emerging clinical problem with the increasing usage of ultrasonography, abdominal computed tomography (CT), and magnetic resonance imaging (MRI) (1). AIs, accounting for approximately 5–7% of the cases in the adult population (2), are mostly nonfunctioning adrenocortical adenomas (AAs) but may also represent conditions requiring further and specific clinical management (e.g., pheochromocytomas [PCCs], adrenocortical carcinoma, or metastasis) (3). Catecholamines are usually produced in PCCs, causing typical symptoms such as hypertension. As is well known, biochemical screening tests of catecholamines and/or catecholamine metabolites play a crucial role in the diagnosis of PCCs. However, these tests may be cumbersome (4) or falsely positive (5). Therefore, imaging examinations, especially CT, are widely used in clinical practice and have an irreplaceable position in the diagnosis of PCCs.

An unenhanced CT attenuation value of > 10 Hounsfield units (HUs) is generally considered as the cutoff value between lipid-rich adenomas (LRAs) and lipid-poor adenomas (LPAs) (6). Washout criteria of contrast material on 15- and 30-min delayed enhanced CT were reported to have a highly specific value for the differentiation of adenomas from non-adenomas (7–9). However, PCCs misdiagnosis as LPAs occurred based on washout criteria in a number of studies (10–12). Hence, no definite approach exists to distinguish PCCs from LPAs in traditional radiological analysis based on unenhanced and delayed enhanced CT scans.

Until now, few studies have explored the value of tri-phasic CT scans in the differentiation of the two lesions (13, 14). Northcutt et al. found that arterial phase enhancement levels higher than 110 HU and lesion heterogeneity facilitated the identification of PCCs (13). Additionally, combining quantitative with qualitative features of tri-phasic CT, An et al. differentiated LPAs from PCCs (14). However, the sample sizes of these studies were relatively small. Furthermore, they were limited to performing integral assessment rather than grouping analysis, in which has limited the application of the technique for accurate diagnosis in clinical practice. Therefore, our study was designed to determine the value of tri-phasic CT images in differentiating PCCs from LPAs using a grouping method based on a relatively large sample. We aimed to establish a risk prediction model for differential diagnosis of the two lesions.

## Materials and methods

### Patients

**Figure 1:**
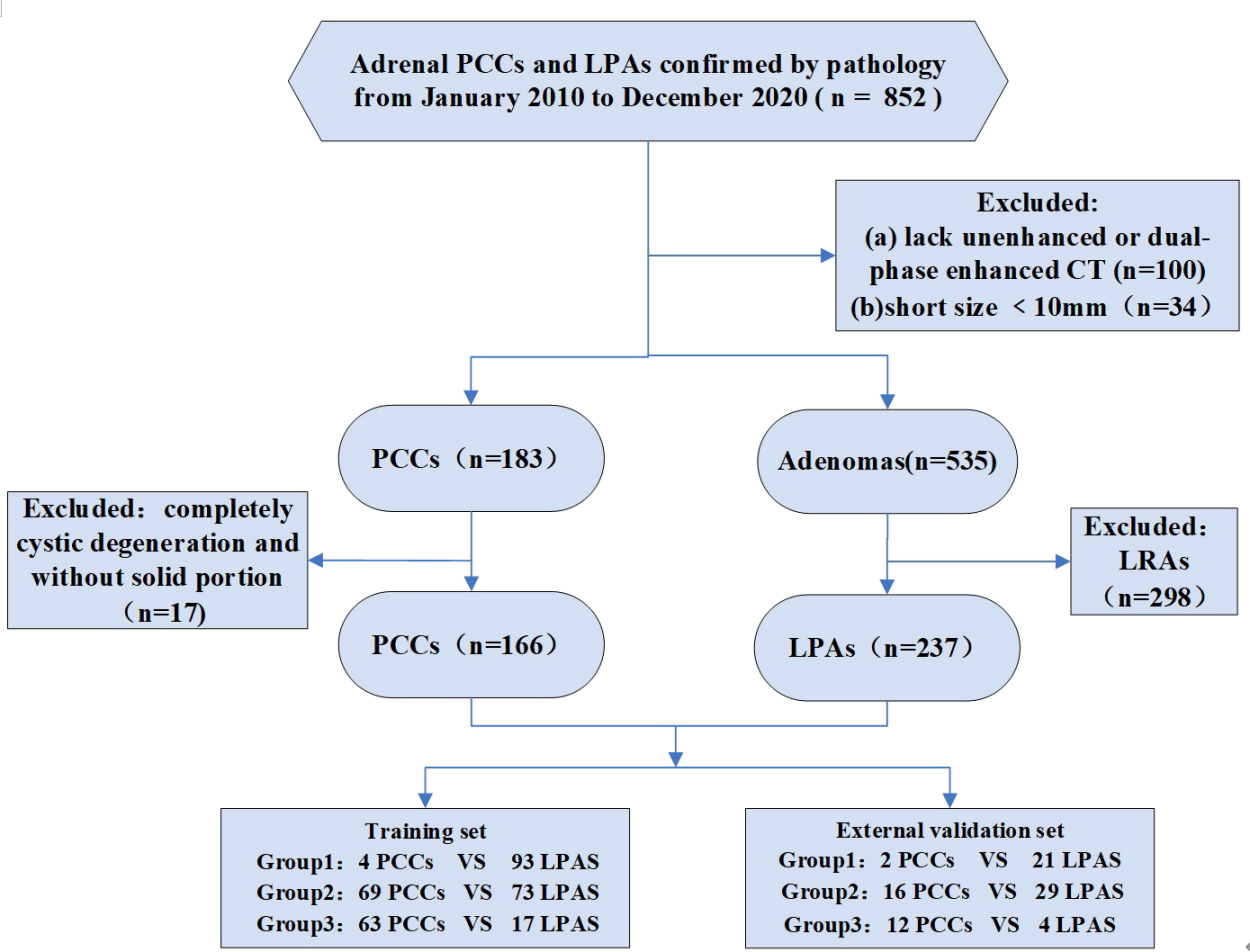
Flowchart shows exclusion criteria for the study. PCCs, pheochromocytomas. LRAs, lipid-rich adenomas. LPAs, lipid-poor adenomas. Group 1: 10 HU < CTu ≤ 25 HU. Group 2: 25 HU < CTu ≤ 40 HU. Group 3: CTu > 40 HU.

This retrospective study was approved by the Institutional Ethical Committees of Sir Run Shaw Hospital and Tongde Hospital of Zhejiang Province, The First Affiliated Hospital of Bengbu Medical College, with waiver of informed consent for participation. Patients’ data were collected by searching the pathology database with the key words “adrenal pheochromocytomas,” “adrenal adenomas.” We found 196 adrenal PCCs and 656 AAs in our search from January 2010 to December 2020. The following inclusion criteria were used: (a) available unenhanced and dual-phase (arterial and venous phase) enhanced CT images and (b) mean unenhanced CT (CTu) attenuation values of AAs were > 10 HU. According to the attenuation values in CTu, lesions were divided into three groups: group 1, 10 HU < CTu ≤ 25 HU; group 2, 25 HU < CTu ≤ 40 HU; and group 3, CTu > 40 HU. This CTu threshold was selected based on the authors’ collected data and pertinent researches on mean CTu values of AAs and PCCs (15, 16); (c) all adrenal PCCs and LPAs were confirmed by pathology results; (d) presence of a solid enhanced region within lesions; (e) a short size ≥ 10 mm was set. Finally, 162 patients with 166 PCCs and 233 patients with 237 LPAs were included in the study. The enrolled patients were assigned to a training set (136 PCCs and 183 LPAs) from Sir Run Shaw Hospital and Tongde Hospital of Zhejiang Province, along with an external independent validation set (30 PCCs and 54 LPAs) from The First Affiliated Hospital of Bengbu Medical College. Hypertension was found in 52.5% (85/162) of the patients with PCCs and in 42.1% (98/233) of those with LPAs. Additionally, almost 4.7% (11/233) of the LPA cases had Cushing’s syndrome, such as weight gain, “moon” facies, and hirsutism. The other patients were without obvious clinical syndromes. The design of this study was retrospective, and biochemical screening test data were thus largely missing. Therefore, this study was focused on CT imaging values, and no evaluation of biochemical screening tests was performed. **Figure 1** is flow chart of the study profile.

### CT image protocols

CT images were obtained using multidetector CT (MDCT) scanners, namely, SOMATOM Sensation 16, SOMATOM Definition Flash (Siemens Healthcare, Forchheim, Germany) and LightSpeed VCT (GE Healthcare, Milwaukee, WI). A total volume of 100–120 ml of contrast material (Ultravist, Bayer Schering Pharma AG, Berlin, Germany) was injected intravenously by a mechanical injector at a rate of 2.5–3.0 ml/sec. Enhanced CT images were obtained during the arterial phase (20–40 sec) and venous phase (60–80 sec) after the infusion of the contrast material. The following CT scan parameters were set up: tube voltage = 120 kVp, tube current = 250–300 mA, detector collimation = 1.5–2.5 mm, table pitch = 1:1, slice thickness = 5 mm. The original images of 5-mm-thick slices were reconstructed into 1.5-mm-thick slices.

### CT image analysis

Two abdominal radiologists (the 1^st^ radiologist had 4 years of experience, and the 2^nd^ radiologist had 15 years of experience.), who were blinded to the pathologic diagnosis, reviewed all existing CT images, respectively and independently. Disagreements about image analyses were resolved by consensus.

The following CT findings were recorded: (a) size: long (LD) and short (SD) diameters; (b) CT attenuation values of unenhanced phase (CTu), arterial phase (CTa), and venous phase (CTv). A circular region of interest (ROI) avoiding the inclusion of peripheral tissues was placed on the enhanced part of the tumor to measure the attenuation value. The area of ROI was larger than 2 cm^2^. These values were measured by the two radiologists and averaged to obtain the final results; (c) the degree of enhancement was calculated as follows: The degree of enhancement in arterial phase (DEap) and venous phase (DEvp) as DEap = CTa – CTu, DEvp = CTv – CTu. DEpeak was determined to be the maximum one of DEap and DEvp; (d) DEpeak was divided by CTu and expressed as an enhancement ratio (ERpeak); (e) absolute percentage washout (APW) = (CTa - CTv) × 100/(CTa - CTu). Relative percentage washout (RPW) = (CTa - CTv) × 100/CTa; (f) the peak enhancement phase (arterial or venous phase) was considered as the phase in which enhancement was maximum with an enhancement level greater than the other one by ≥ 5 HU. If the level was < 5 HU different, it was determined to be equally enhanced; (g) location: the left or right adrenal gland; (h) shape: round/oval (the LD/SD ratio ≤ 1.2) and not round/oval (the LD/SD ratio > 1.2); (i) the presence of calcification, which CTu was more than 100 HU; (j) the presence of cystic degeneration, with CTu of low attenuation area less than 20 HU, as well as DEap and DEvp all less than 10 HU; (k) the presence of hemorrhage on CTu was between 55 and 90 HU and without enhancement; (l) the presence of an intratumoral vessel, which was observed on arterial phase images.

### Statistical analysis

All statistical data analyses were performed with SPSS (version 25.0, SPSS, Chicago, IL). Statistical significance was considered at *P* < 0.05. Continuous data are expressed as mean ± standard deviation or median value (from the 25^th^ to the 75^th^ percentile), whereas categorical variables are expressed as a proportion. For continuous data, independent *t*-test was used for comparison if the data were normally distributed; otherwise, Mann-Whitney *U* test was implemented. For categorical variables, Pearson or continuity correction chi-square test was applied. A receiver operating curve (ROC) was utilized to determine the cutoff values of the maximum sensitivity and specificity obtained at the maximal value of the Youden’s index. Subsequently, statistical significance variables on univariate analysis were included as independent factors in the logistic regression analysis to establish a prediction model. External validation (AUC, accuracy, sensitivity, and specificity) was performed to evaluate the model’s performance.

## Results

### Univariate analyses of the demographic and imaging characteristics

The results of the training set are displayed in **Tables 1** **–** **3**. Additional data of the external validation set are presented in **Supplementary Materials**. The numbers of PCCs and LPAs in each group of the training set are shown in these tables. As can be seen from these tables, CTu values of all PCCs were greater than 10 HU. 2.9% (4/136) of the PCCs had CTu values within 10–25 HU (group 1), whereas 50.7% (69/136) of the PCCs had CTu values within 25–40 HU (group 2), and 46.4% (63/136) of the PCCs had CTu values higher than 40 HU (group 3).

**Table 1:** Demographic characteristics between adrenal PCCs and LPAs on the training set.

	Group 1		*P*-value	Group 2		*P*-value	Group 3		*P*-value	All subjects (groups 1, 2, 3)		*P*-value
	PCCs (*n* = 4)	LPAs (*n* = 93)		PCCs (*n* = 69)	LPAs (*n* = 73)		PCCs (*n* = 63)	LPAs (*n* = 17)		PCCs (*n* = 136)	LPAs (*n* = 183)	
**Age**	53.50 ± 13.67	51.77 ± 11.47	0.770*	49.77 ± 14.38	52.93 ± 10.72	0.142*	50.71 ± 12.77	47.59 ± 12.26	0.370*	50.12 ± 13.67	51.85 ± 11.28	0.231*
**Gender**												
Men	2 (50)	39 (42.9)	1.000^▼^	33 (47.8)	28 (38.4)	0.255^▼^	30 (50.8)	8 (53.3)	0.836^▼^	65 (49.2)	75 (41.9)	0.198^▼^
Women	2 (50)	52 (57.1)		36 (52.2)	45 (61.6)		29 (49.2)	7 (46.7)		67 (50.7)	104 (58.1)	

Numbers of lesions in each group are indicated in brackets. *Data that conform to a normal distribution are means ± standard deviation, and the statistical values are the independent sample t-test results.▼Data in parentheses are percentages, and the statistical values are Pearson or continuity correction chi-square test results. PCCs, pheochromocytomas. LPAs, lipid-poor adenomas.

The demographic characteristics are shown in **Table 1**. No significant difference was found between adrenal PCCs and LPAs of overall groups with patients’ age. Regarding gender, no significant difference was found in all of these groups.

Quantitative CT features of the adrenal PCCs and LPAs in the different groups are shown in **Table 2**. The difference in CTu value between PCCs and LPAs was statistically significant in group 1. Compared with LPAs, PCCs were significant greater in both LD and SD values in group 3. Significant differences were observed in the CTu, CTa, CTv, DEap, DEpeak, ERpeak, LD, and SD values in group 2 and all subjects (groups 1, 2, and 3). APW and RPW values also showed statistically significant differences in all subjects (groups 1, 2, and 3).

**Table 2:** Quantitative CT features between adrenal PCCs and LPAs on the training set.

	Group 1		P-value	Group 2		P-value	Group 3		P-value	All subjects (groups 1, 2, 3)		P-value
	PCCs (n = 4)	LPAs (n = 93)		PCCs (n = 69)	LPAs (n = 73)		PCCs (n = 63)	LPAs (n = 17)		PCCs (n = 136)	LPAs (n = 183)	
**Attenuation**												
CTu	20.50 ± 1.54	17.16 ± 4.24	0.012*	35.60(31.6,38.2)	31.70(27.9,35.8)	**0.001†**	44.5(42.8,47.7)	42.8(41.1,47.5)	0.797†	39.30(34.7,43.9)	24.90(17.0,33.7)	**0.000†**
CTa	48.70(39.1,69.8)	51.40(40.3,63.0)	0.814†	102.93 ± 47.88	75.37 ± 21.769	0.000*	96.72 ± 39.18	92.74 ± 32.47	0.702*	87.4(63.6,126.7)	62.20(48.5,77.8)	**0.000†**
CTv	52.80(42.4,92.9)	63.60(54.4,79.4)	0.384†	105.03 ± 36.53	82.89 ± 17.450	0.000*	95.28 ± 25.31	100.82 ± 21.89	0.413*	99.27 ± 32.38	76.64 ± 22.808	0.000*
**Degree of enhancement**												
DEap	26.90(19.6,49.7)	34.00(24.4,45.6)	0.525†	68.31 ± 47.99	43.26 ± 21.09	0.000*	51.54 ± 40.03	47.08 ± 32.82	0.674*	46.45(25.2,84.3)	37.40(25.6,50.3)	**0.003†**
DEvp	32.45(22.9,71.2)	46.60(35.4,60.9)	0.249†	70.41 ± 36.98	50.78 ± 16.84	0.000*	50.11 ± 26.13	55.17 ± 23.99	0.474*	51.20(34.2,79.6)	50.10(36.4,61.6)	0.143†
DEpeak	48.00(37.5,75.5)	50.70(40.5,61.9)	0.993†	81.80 ± 43.41	55.52 ± 16.68	0.000*	61.21 ± 37.35	61.57 ± 26.29	0.970*	60.70(40.0,95.8)	52.10(43.2,65.8)	**0.009†**
ERpeak	2.45 (1.9,3.6)	3.00 (2.3,4.2)	0.314†	2.10 (1.3,3.4)	1.70 (1.4,2.1)	**0.016†**	1.38 ± 0.88	1.40 ± 0.59	0.955*	1.60(1.0,2.7)	2.20(1.60,3.10)	**0.000†**
APW	-50.60(-152.9,34.6)	-51.08(-112.9,6.7)	0.882†	-11.45(-92.9,24.6)	-14.09(-70.8,13.2)	0.931†	-14.75(61.3,25. 7)	-18.28(-156.6, 19.1)	0.293†	-15.74(-74.9,24.6)	-25.25 (-95.4, 11.3)	**0.043†**
RPW	-25.93(-86.6,28.6)	-30.59(-67.6,4.5)	0.786†	-5.89(-35.6,17.3)	-6.21(-36.8,8.5)	0.642†	-7.04 (22.2,17. 4)	-7.07(-52.2,10. 8)	0.514†	-7.47 (-26.6,17,1)	-15,89(-53.4,7.2)	**0.002†**
**Size**												
LD	26.50(12.8,45.5)	22.00(16.5,27.5)	0.561†	36.00(25.5,46.0)	21.00(17.5,26.5)	**0.000†**	38.00 (27.0, 55. 0)	22.00(16.0,35.0)	**0.022†**	36.00(36.3,49.0)	22.00(17.0,27.0)	**0.000†**
SD	21.50(11.8,36.5)	17.00(13.0,22.0)	0.462†	30.00(22.0,40.5)	17.00(14.0,22.0)	**0.000†**	33.00 (23.0, 46. 0)	19.00(12.5,27.5)	**0.018†**	30.00(23.0,42.0)	17.00(13.0,22.0)	**0.000†**
LD/SD	1.23 (1.1,1.3)	1.23 (1.1,1.4)	0.479†	1.16 (1.1,1.3)	1.18 (1.1,1.3)	0.705†	1.22 ± 0.19	1.21 ± 0.11	0.944*	1.17 (1.1,1.3)	1.20 (1.1,1.4)	0.073†

Each group’s tumor number is shown in brackets. *Data with normal distribution are means ± standard deviation, and independent sample t-tests provide P-values. †Data that do not conform to a normal distribution are expressed as the median value (from the 25^th^ to 75^th^ percentile), and the statistical values are Mann-Whitney U test results. P-values written in bold indicate a significant difference between lesions. PCCs, pheochromocytomas. LPAs, Lipid-poor adenomas. CTu/CTa/CTv, the CT attenuation value of unenhanced/arterial/venous phase. DEap, CTa – CTu. DEvp, CTv – CTu. ERpeak, DEpeak/CTu. DEpeak is the peak value between DEap and DEvp. APW = (CTa - CTv) × 100/(CTa - CTu). RPW, (CTa - CTv) × 100/CTa. LD, the long diameter. SD, the short diameter.

Qualitative CT features are presented in **Table 3**. There were significant differences of cystic degeneration between PCCs and LPAs in all of these groups. In addition, intratumoral vessel and hemorrhage were significant different in all subjects (groups 1, 2, and 3). Intratumoral vessel was also significant different in group 2. A statistically significant difference was also observed in location in group 3.

**Table 3:** Qualitative CT features between adrenal PCCs and LPAs on the training set.

	Group 1		*P*-value	Group 2		*P*-value	Group 3		*P*-value	All subjects (groups 1, 2, 3)			*P*-value
	PCCs (n = 4)	LPAs (n = 93)		PCCs (n = 69)	LPAs (n = 73)		PCCs (n = 63)	LPAs (n = 17)		PCCs (n = 136)		LPAs (n = 183)	
**Location**													
Right	2 (50)	47(50.5)	1.000	37(53.6)	34(46.6)	0.401	20 (31.7)	10 (58.8)	**0.041**	59(43.4)		91(49.7)	0.262
Left	2 (50)	46(49.5)		32(46.4)	39 (53.4)		43(68.3)	7(41.2)		77(56.6)		92(50.3)	
**Shape**													
Round/oval	2 (50)	38 (40.9)	1.000	38(55.1)	46(63.0)	0.336	29 (46.0)	7 (41.2)	0.721	77(56.6)		90(49.2)	0.188
Not round/oval	2 (50)	55 (59.1)		31 (44.9)	27(37.0)		34(54.0)	10(58.8)		59(43.4)		93(50.8)	
**Peak enhanced phase**													
Arterial phase	1(25)	27(29.0)	0.842	28(40.6)	38(52.0)	0.072	24(38.1)	6(35.3)	0.753	53(39.0)		71(38.8)	0.390
Venous phase	3(75)	60(64.5)		39(56.5)	28(38.4)		35(55.6)	9(52.9)		77(56.6)		97(53.0)	
Equally enhanced	0	6(6.5)		2(2.9)	7(9.6)		4(6.3)	2(11.8)		6(4.4)		15(8.2)	
**Calcification**	1 (25)	5 (5.4)	0.592	5(7.2)	3(4.1)	0.418	6(9.5)	2(11.8)	0.785	12(8.8)		10(5.5)	0.242
**Cystic degeneration**	2 (50)	6 (6.5)	**0.030**	50(72.5)	3(4.1)	**0.000**	48(76.2)	1 (5.9)	**0.000**	100(73.5)		10(5.5)	**0.000**
**Hemorrhage**	0(-)	2(2.2)	1.000	3(4.3)	1(1.4)	0.284	9(14.3)	1(5.9)	0.353	12(8.8)		4(2.2)	**0.007**
**Intratumoral vessel**	1(25)	4(4.3)	0.497	19(27.5)	6 (8.2)	**0.003**	22(34.9)	4(23.5)	0.374		42(30.9)	14(7.7)	**0.000**

Data are numbers of lesions. Data in parentheses are percentages. There is a significant difference when a P-value is in bold.

PCCs, pheochromocytomas. LPAs, lipid-poor adenomas.

### ROC analyses of the significant independent variables

**Figure 2:**
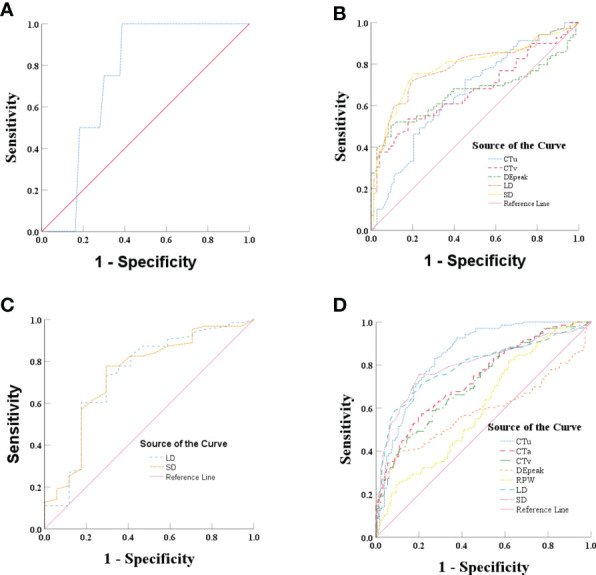
**(A)** Graph shows ROC of CTu for differentiating PCCs from LPAs in group 1. **(B)** Graph shows ROCs of CTu, CTv, DEpeak, LD, and SD in group 2. **(C)** Graph shows ROCs of LD and SD in group 3. **(D)** Graph shows ROCs of CTu, CTa, CTv, DEpeak, RPW, LD, and SD in all subjects (groups 1, 2, and 3).

ROC analysis was performed of the quantitative variables, which had shown significant differences in the univariate analysis. In group 1, ROC curve was analyzed for CTu value, using ≥ 19.55 HU as the cutoff value for distinguishing PCCs from LPAs (**Figure 2A**). In group 2, we established that SD (≥ 24.50 mm), LD (≥ 28.50 mm), CTv (≥ 87.80 HU), DEpeak (≥ 58.95 HU), and CTu (≥ 33.45 HU) were independent predictive factors for identifying PCCs (**Figure 2B**). In group 3, an analysis of ROC curve was analyzed for LD and SD values, indicating that the cutoff values of LD and SD were ≥ 28.50 and ≥ 22.50 mm, respectively (**Figure 2C**).

In all subjects (groups 1, 2, and 3), ROC analysis was performed by using the same approach. The results showed that CTu (≥ 35.55 HU), SD (≥ 23.50 mm), LD (≥ 24.50 HU), CTa (≥ 68.25 HU), CTv (≥ 83.35 HU), DEpeak (≥ 51.35 HU), RPW (≥ −23.00) were independent predictive factors for distinguishing PCCs from LPAs (**Figure 2D**).

### Binary logistic regression analysis and prediction model establishment

Based on the univariate analysis result, two variables (CTu and cystic degeneration) and four variables (LD, SD, location, and cystic degeneration) were involved in the binary logistic regression analysis in groups 1 and 3, respectively. Finally, statistical significance was only found in cystic degeneration for distinguishing PCCs from LPAs in above two groups.

**Figure 3:**
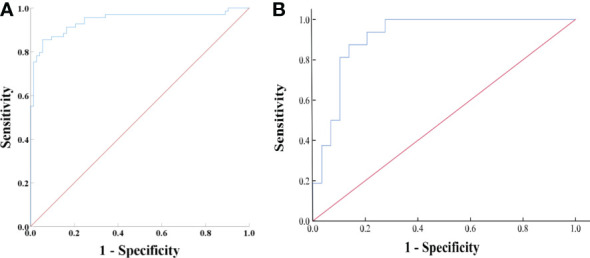
**(A)** ROC analysis of the model in group 2 on the training set. The AUC was 0.94 (95% CI: 0.902–0.984), with accuracy, sensitivity, specificity of 88.7, 90.4, and 87.0%, respectively. **(B)** ROC analysis of the model in group 2 on the external validation set. The AUC was 0.91 (95% CI, 0.823–0.927) with accuracy, sensitivity, and specificity of 82.2, 69.0, and 90.0%, respectively.

Based on the univariate and ROC analyses results, in group 2, seven variables—SD, LD, CTv, DEpeak, CTu, cystic degeneration, and intratumoral vessel—were involved in the binary logistic regression analysis. Finally, three variables—cystic degeneration, CTu ≥ 33.45 HU, and DEpeak ≥ 58.95 HU—were found to have statistical significance (**Table 4**). According to the coefficients obtained from the aforementioned analysis, the following prediction equation for PCCs was derived as follows: *Y* = −8.991 + 4.873*(cystic degeneration) + 0.151*(CTu ≥ 33.45 HU) + 0.062*(DEpeak ≥ 58.95 HU). ROC curves were drawn with an AUC of 0.94 [95% confidence interval (CI): 0.902–0.984] in the training set (**Figure 3A**) and 0.91 (95% CI, 0.823–0.927) in the external validation set (**Figure 3B**).

**Table 4:** Binary regression analysis for identifying adrenal PCCs in group 2 on the training set.

Variables	OR	B	Wald X^2^-value	*P*-value	95% CI
**Cystic degeneration**	130.741	4.873	26.213	**0.000**	20.240–844.510
**CTu**	1.162	0.151	4.077	**0.043**	1.004–1.345
**DEpeak**	1.064	0.062	8.411	**0.004**	1.020–1.109
Intratumoral vessel	2.207	0.792	1.017	0.313	0.474–10.287
LD	1.077	0.074	0.703	0.402	0.905–1.282
CTv	0.979	-0.021	1.020	0.312	0.940–1.020
SD	0.921	-0.082	0.568	0.451	0.744–1.141
Constant	0.000	-8.991	10.618	0.001	—————

PCCs: Pheochromocytomas. CTu/CTv, the CT attenuation value of unenhanced/venous phase. DEpeak is the peak value between DEap and DEvp. LD, the long diameter. SD, the short diameter. Bold values indicate statistically significant differences.

**Figure 4:**
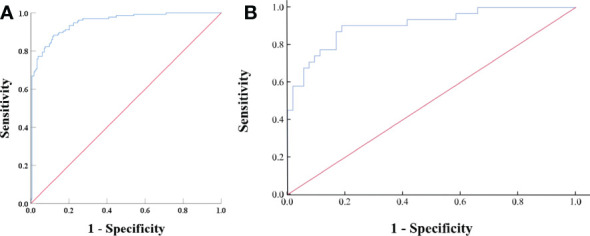
**(A)** ROC analysis of the model in all subjects (groups 1, 2, and 3) on the training set. The value of the AUC was 0.95 (95% CI: 0.927–0.973). The accuracy, sensitivity, and specificity of the model were 87.1, 91.2, and 82.0%, respectively. **(B)** ROC analysis of the model in all subjects (groups 1, 2, and 3) on the external validation set. The AUC was 0.91 (95% CI, 0.860–0.929), with accuracy, sensitivity, and specificity of 80.9, 64.5, and 90.6%, respectively.

**Figure 5:**
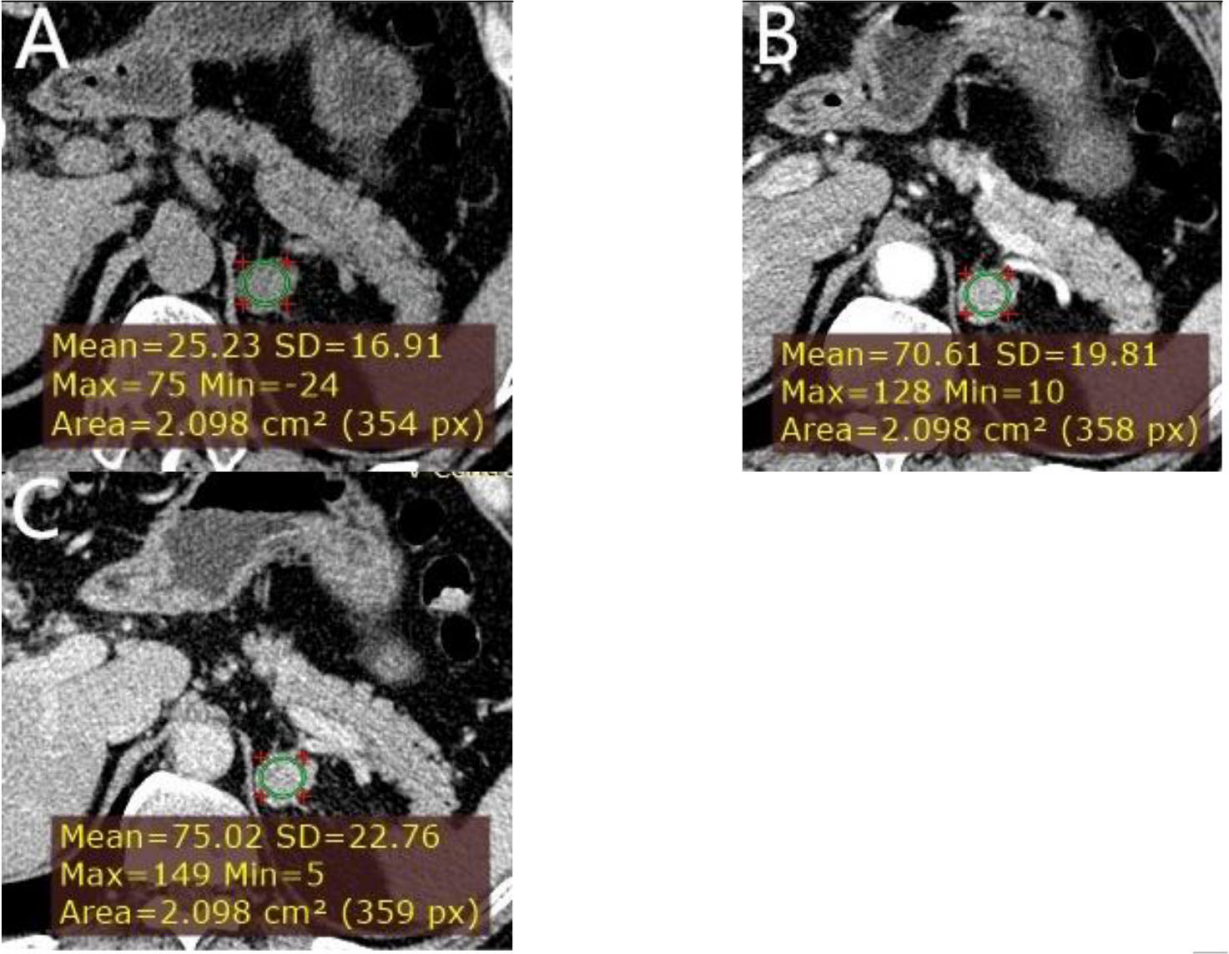
** **A left adrenal LPA in a 63-year-old man. **(A)** Axial unenhanced phase. **(B)** Axial arterial phase. **(C)** Axial venous phase. The values of CTu, CTa, CTv, and DEpeak were 25.23, 70.61, 75.02, and 49.79 HU, respectively. LD and SD of the mass were 25 and 20 mm. No cystic degeneration was seen within the tumor.

**Figure 6:**
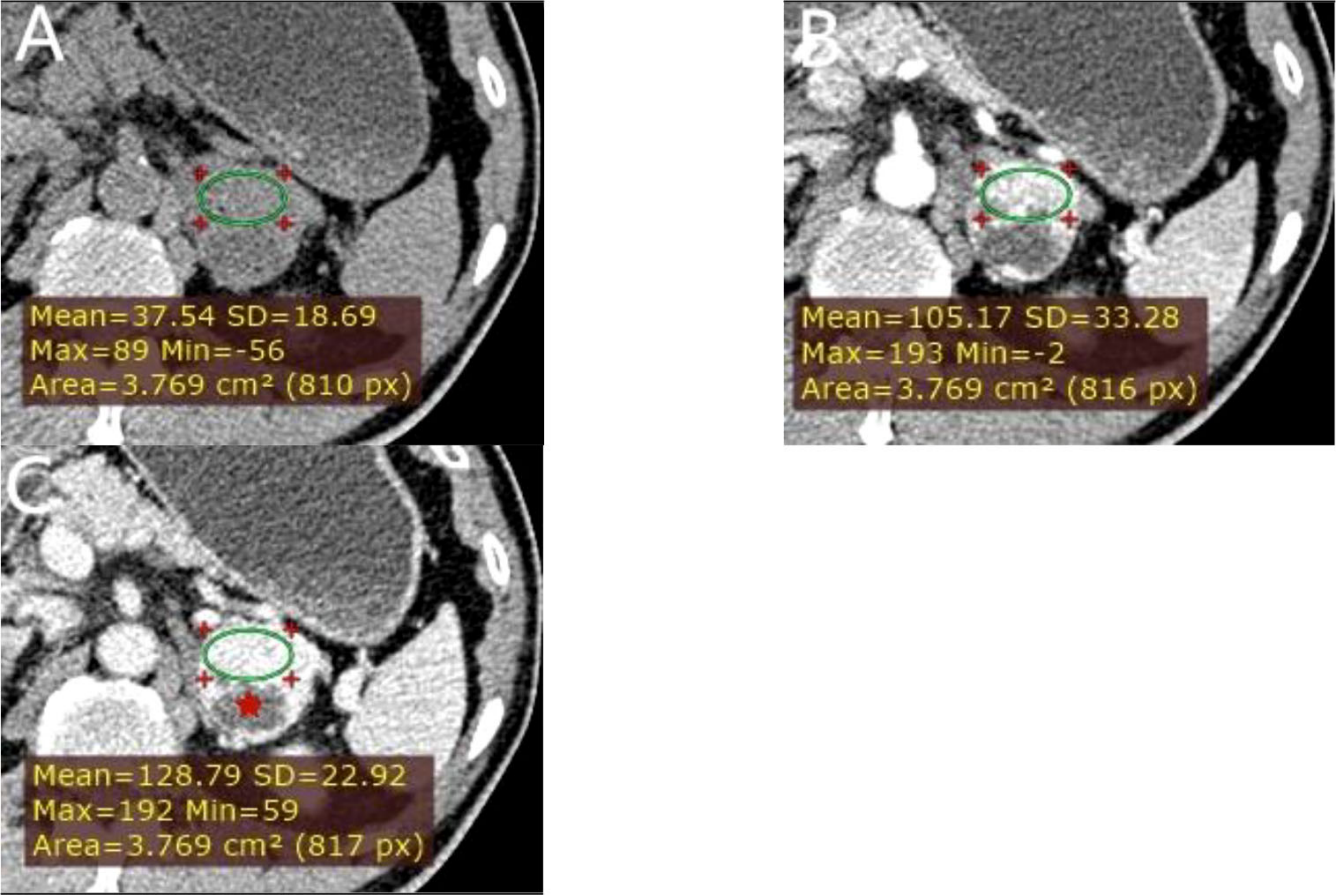
** **A left adrenal PCC in a 57-year-old man. **(A)** Axial unenhanced phase. **(B)** Axial arterial phase. **(C)** Axial venous phase. The values of CTu, CTa, CTv, and DEpeak were 37.54, 105.17, 128.79, and 91.25 HU, respectively. LD and SD of the mass were 40 and 38 mm. Cystic degeneration was seen within the Lesion (red star).

Based on the results of the univariate and ROC analyses in all subjects (groups 1, 2, and 3), 10 variables—CTu, SD, LD, CTa, CTv, DEpeak, RPW, cystic degeneration, hemorrhage, and intratumoral vessel were involved in the binary logistic regression analysis. Finally, statistical significance was established for three variables—cystic degeneration, CTu ≥ 35.55 HU, and DEpeak ≥ 51.35 HU (**Table 5**). According to the coefficients obtained in the above analysis, the following prediction equation for PCCs was derived: *Y* = −7.709 + 3.617*(cystic degeneration) + 0.175*(CTu ≥ 35.55 HU) + 0.068*(DEpeak ≥ 51.35 HU). The model demonstrated a great predictive efficacy with an AUC of 0.95 (95% CI: 0.927–0.973; training set) and 0.91 (95% CI: 0.860–0.929; external validation set) (**Figures 4A, B**). To illustrate better the adopted imaging characteristics, the images of clinical examples of patients with a PCC and LPA are displayed in **Figures 5**, **6**.

**Table 5:** Binary regression analysis for identifying adrenal PCCs in all subjects (groups 1, 2, and 3) on the training set.

Variables	OR	B	Wald X^2^-value	*P*-value	95% CI
**Cystic degeneration**	37.223	3.617	42.923	**0.000**	12.615–109.833
**CTu**	1.191	0.175	18.413	**0.000**	1.100–1.291
**DEpeak**	1.071	0.068	4.744	**0.029**	1.007–1.138
Intratumoral vessel	1.328	0.284	0.302	0.583	0.482–3.659
RPW	1.017	0.017	0.656	0.418	0.976–1.060
SD	1.044	0.043	0.376	0.540	0.909–1.200
LD	0.985	-0.015	0.074	0.785	0.882–1.100
CTa	0.970	-0.031	0.628	0.428	0.898–1.047
CTv	0.982	-0.018	0.624	0.430	0.939–1.027
Hemorrhage	0.333	-1.101	1.598	0.206	0.060–1.832
Constant	0.000	-7.709	49.815	0.000	————–

PCCs, Pheochromocytomas. CTu/CTa/CTv, the CT attenuation value of unenhanced/arterial/venous phase. DEpeak is the peak value between DEap and DEvp. RPW = (CTa - CTv) × 100/CTa. LD, the long diameter. SD, the short diameter. Statistics indicate that the bolded values are statistically significant.

## Discussion

A prediction model for identifying PCCs from LPAs based on tri-phasic CT image features was established in this study. Cystic degeneration, CTu, and DEpeak were independent risk factors for the differentiation. We established good performance for the differentiation of the model with an AUC of 0.95 (training set) and 0.91 (external validation set). Our study achieved an accurate diagnosis using a grouping method based on a relatively large sample. These results of our study may serve as a foundation for precision diagnosis.

Cystic degeneration was significant in all groups, which was more prevalent in PCCs (73.5%) than in LPAs (5.5%). The frequency of cystic degeneration in PCCs was greater than the one established in an earlier study (17) but that in LPAs was consistent with this previous evidence. The biggest discrepancy was the scanning time after contrast injection (15 min versus almost 1 min). That study (17) deemed cystic degeneration as low hypodense area persistently seen at the same level on both 1- and 15-min enhanced CT. However, we considered cystic degeneration as area of low attenuation increased its attenuation by 0–10 HU on almost 1-min enhanced CT. Our findings support the notion that heterogeneity was a feature associated with PCCs and not common in LPAs, which was consistent with the findings of an earlier investigation (18).

The value of DEpeak was one of the independent predictive factors for distinguishing PCCs from LPAs. Nagayama et al. (19) reported that a relative enhancement ratio of the venous phase to unenhanced CT ≥ 210% accurately differentiated LPAs from non-adenomas. In another research, Goroshi et al. (20) found that a percentage arterial enhancement value ≥ 100% differentiated PCC from other malignant adrenal masses with high specificity. Nevertheless, we used the DEpeak value to evaluate the degree of enhancement in our study. DEpeak is the maximum value between the degree of enhancement of the arterial phase (DEap) and the venous phase (DEvp), which implies it has the best performance. We consider that it was not only related to cluster 1 (possible peak enhancement on arterial phase) versus cluster 2 (possible peak enhancement on venous phase) but also the value of CTu.

The value of CTu was another independent predictive factor for distinguishing PCCs from LPAs. In our study, PCCs usually had CTu values of greater than 40 HU, but more often, between 25 and 40 HU. Furthermore, it was noteworthy that no PCC had a lower than 10 HU CTu value. However, the probability that the lesion might have been a PCC was low but not zero if the CTu value of the lesion was between 10 HU and 25 HU. Our findings were generally in accordance with those obtained in the study of Canu et al. (21), who found no PCCs with CTu value of less than 10 HU and that only 0.5% of PCCs had CTu values of equal to 10 HU.

Based on the aforementioned, we can make the following conclusions. First, if the CTu value of a lesion in incidentally discovered adrenal masses in clinic practice is less than 10 HU, biochemical test is not required to exclude a PCC. Our viewpoint is in line with those of previous studies (21, 22). Nevertheless, if a CTu value of a lesion is greater than 10 HU, especially greater than 25 HU, further biochemical test for PCCs in AIs is to be inevitable. That is particularly valid when incidentally discovered adrenal masses exhibited these tri-phasic CT image features (cystic degeneration, CTu ≥ 35.55 HU, and DEpeak ≥ 51.35 HU), which highly suggests diagnosis of PCCs. Although the probabilities of PCCs in each of these groups of our study were not low enough to avoid biochemical tests, we provided a mean to predict the occurrence of PCCs before biochemical tests.

Through an extensive systematic search of relevant publications, we have developed the first prediction model for identifying PCCs from LPAs using the grouping method based on a relatively large sample. An earlier study (23) also established a predictive model for the distinction of PCCs from non−PCCs, which consisted of LRAs (CTu < 10 HU). However, our study excluded LRAs, because CTu < 10 HU on an unenhanced CT scan image was reliable to diagnose LRAs. Two points may be worthy of attention concerning the clinical applicability of our model. First, our study was setting defined by multicenter with varied protocols of the CT scanners, which could account for heterogeneity from center to center in different instruments used for CT. Second, the model plays an important role in the differentiation between PCCs and LPAs in clinical practice, particularly with relevance to any associated need for biochemical tests for diagnosis PCCs in AIs.

Nevertheless, the present investigation has some limitations that need to be addressed in future studies. First, discrepancies in the measurements and analyses performed by the two radiologists might have occurred. However, these measurement values were measured and averaged as definitive results to reduce differences. Second, cystic degeneration in PCCs was found to be much more frequent in our study than in previous ones. This finding may limit the generalizability of the model. Third, due to the retrospective nature of this research, most of the biochemical screening tests data were missing. However, the study aimed to analyze the CT imaging features of PCCs and LPAs. Our investigation also has a notable strength as it clearly confirmed the effectiveness and applicability of the predictive model validated by external test set.

In conclusion, a reliable and practical prediction model for differential diagnosis of adrenal PCCs and LPAs was established in the study. In addition, a novel method was provided to predict the occurrence of PCCs before biochemical tests based on the prediction model suggested. For a lesion in AIs with CTu value less than 10 HU, biochemical test for PCCs is not required. Nevertheless, if the CTu value of the lesion is higher than 10 HU, further biochemical test for PCCs should be inevitable. Especially when imaging features, including cystic degeneration, CTu ≥ 35.55 HU, and DEpeak ≥ 51.35 HU, are present in tri-phasic CT scans, it highly suggests diagnosis of PCCs. In such situations, application of tri-phasic CT scans combined with biochemical screening tests should be useful for confirmed diagnosis of PCCs by clinicians.

## Data availability statement

The raw data supporting the conclusions of this article will be made available by the authors, without undue reservation.

## Ethics statement

The study was approved by the institutional review board of Sir Run Run Shaw Hospital, Zhejiang University School of Medicine. Written informed consent was waived by the Institutional Review Board. Written informed consent for participation was not required for this study in accordance with the national legislation and the institutional requirements.

## Author contributions

Conceptualization, methodology: ZN, JW, FZ. Formal analysis, investigation, resources, data curation: ZN, JW, YY, JH, SW, ZX, MS and FZ. Writing -original draft preparation: ZN, JW. Writing - review and editing: ZN, JW, FZ. Visualization, supervision, project administration: FZ. All authors contributed to the article and approved the submitted version.
