# Supplementary material for: Risk prediction model establishment with tri-phasic CT image features for differential diagnosis of adrenal pheochromocytomas and lipid-poor adenomas: Grouping method
Source: Front Endocrinol (Lausanne). 2022 Dec 8;13:925577. doi: 10.3389/fendo.2022.925577 (PMC9772429; doi:10.3389/fendo.2022.925577)
Supplement: Supplementary file 1 [file Table_1.docx]

**Supplementary table 1.** Demographic characteristics and quantitative CT features between adrenal PCCs and LPAs on the external validation set.

|  | **Group 2**  **_________________________________________________________**  **PCCs (n = 16) LPAs (n = 29)** | | ***P*-value** | **All subjects (groups 1, 2, 3)**  **__________________________________________________**  **PCCs (n = 30) LPAs (n = 54)** | | ***P*-value** |
| --- | --- | --- | --- | --- | --- | --- |
| Age | 51.69 ± 11.82 | 48.90±10.40 | 0.416* | 54.07±11.905 | 50.43±11.539 | 0.174* |
| **Attenuation** | | | | | | |
| CTu | 36.65(34.08, 37.85) | 30.40(27.35, 34.75) | **0.001†** | 38.30(35.35, 42.05) | 26.65(18.73, 34.10) | **0.000†** |
| CTa | 89.00(64.59, 121.38) | 74.50(57.90, 86.45) | **0.045†** | 87.15(71.15, 101.73) | 66.30(54.63, 79.70) | **0.000†** |
| CTv | 95.45(79.60, 127.55) | 92.00(79.15, 103.40) | 0.522† | 95.45(84.00, 107.14) | 79.80(71.33, 96.85) | **0.025†** |
| **Degree of enhancement** | | | | | | |
| DEap | 51.30(31.95, 82.90) | 40.40(29.60, 51.60) | 0.200† | 47.85(28.05, 67.07) | 40.35(27.30, 51.10) | 0.169† |
| DEvp | 58.05(40.65, 91.68) | 64.50(47.00, 70.70) | 0.749† | 52.75(39.83, 72.75) | 58.15(47.05, 69.53) | 0.535† |
| DEpeak | 59.95(49.73, 103.53) | 64.80(52.75, 74.10) | 0.981† | 58.58(47.73, 85.50) | 59.50(50.60, 70.30) | 0.837† |
| ERpeak | 1.61(1.31, 2.88) | 2.13(2.49, 1.71) | 0.319† | 1.49(1.08, 2.29) | 2.40(1.79, 3.16) | **0.002†** |
| APW | 2.73(-26.14, 15.25) | -60.62(-103.01, 5.17) | **0.035†** | -7.24(-41.64, 11.79) | -49.74(-97.65, 0.60) | **0.017†** |
| RPW | 1.55(-15.03, 8.52) | -32.60(-49.79, 2.95) | **0.035†** | -3.42(-23.64, 8.46) | -31.12(-48.50, 0.14) | **0.006†** |
| **Size** | | | | | | |
| LD | 45.95(31.18, 61.30) | 20.40(15.75, 24.50) | **0.001†** | 39.75(23.13, 54.10) | 19.20(14.73, 24.65) | **0.000†** |
| SD | 35.55(25.52, 44.53) | 15.60(12.65, 18.60) | **0.000†** | 32.25(19.38, 43.50) | 15.20(11.98, 20.30) | **0.000†** |
| LD/SD | 1.18(1.08, 1.18) | 1.27(1.07, 1.38) | 0.619† | 1.17(1.08, 1.33) | 1.27(1.07, 1.40) | 0.375† |

Note.—*Data that conform to a normal distribution are means ± standard deviation, and the statistical values are the independent sample t-test results. †Data that do not conform to a normal distribution are expressed as the median value (from the 25^th^ to 75^th^ percentile), and the statistical values are Mann-Whitney U test results. *P*-values written in bold indicate a significant difference. PCCs: Pheochromocytomas. LPAs: Lipid-poor adenomas. CTu/CTa/CTv = the CT attenuation value of unenhanced /arterial /venous phase. DEap = CTa – CTu. DEvp = CTv – CTu. ERpeak = DEpeak/CTu. DEpeak is the [peak](javascript:void(0);) value between DEap and DEvp. APW = (CTa - CTv) × 100/(CTa - CTu). RPW = (CTa - CTv) × 100/CTa. LD = the long diameter. SD = the short diameter.

**Supplementary table 2.** Demographic characteristics and qualitative CT features between adrenal PCCs and LPAs on the external validation set.

|  | **Group 2** | | ***P*-value** | **All subjects（groups 1, 2, 3）** | | ***P*-value** |  |  |
| --- | --- | --- | --- | --- | --- | --- | --- | --- |
|  | **PCCs (n = 16)** | **LPAs (n = 29)** |  | **PCCs (n = 30)** | **LPAs (n = 54)** |  |  |  |
| **Gender** | | | | | | | |  |
| Men | 10(62.5) | 14(48.3) | 0.486 | 13(43.3) | 28(51.9) | 0.454 |  |  |
| Women | 6(37.5) | 15(51.7) |  | 17(56.7) | 26(48.1) |  |  |  |
| **Location** | | | | | | | |  |
| Right  Left  **Shape** | 8(50)  8(50) | 11(37.9)  18(62.1) | 0.433 | 13(43.3)  17(56.7) | 20(37.0)  34(63.0) | 0.571 |  |  |
| Round/oval  Not round/oval  **Peak enhanced phase**  Arterial phase  Venous phase  Equally enhanced | 8(50)  8(50)  5(31.3)  5(31.3)  6(37.4) | 11(37.9)  18(62.1)  6(20.7)  20(68.9)  3(10.4) | 0.433  **0.037** | 16(53.3)  14(46.7)  8(26.7)  14(46.7)  8(26.6) | 22(40.7)  32(59.3)  9(16.7)  37(68.5)  8(14.8) | 0.267  0.146 |  |  |
| **Calcification** | 5(31.3) | 0(-) | **0.004** | 12(40.0) | 10(18.5) | **0.020** |  |  |
| **Cystic degeneration** | 10(62.5) | 1(3.4) | **0.000** | 5(16.7) | 1(1.85) | **0.000** |  |  |
| **Hemorrhage** | 0(-) | 0(-) | ------- | 1(3.3) | 0(-) | 0.357 |  |  |
| **Intratumoral vessel** | 1(6.25) | 2(6.9) | 1.000 | 1(3.3) | 4(7.4) | 0.651 |  |  |

Note.—Data are numbers of lesions. Data in parentheses are percentages. Statistics indicate that the bolded values are statistically significant.

PCCs: Pheochromocytomas. LPAs: Lipid-poor adenomas.
